# Plant Bioactives and the Prevention of Prostate Cancer: Evidence from Human Studies

**DOI:** 10.3390/nu11092245

**Published:** 2019-09-18

**Authors:** Tracey L. Livingstone, Gemma Beasy, Robert D. Mills, Jenny Plumb, Paul W. Needs, Richard Mithen, Maria H. Traka

**Affiliations:** 1 Quadram Institute Bioscience, Norwich Research Park, Norwich, Norfolk NR4 7UQ, UK; tracey.livingstone@quadram.ac.uk (T.L.L.); jenny.plumb@quadram.ac.uk (J.P.); paul.needs@quadram.ac.uk (P.W.N.); richard.mithen@quadram.ac.uk (R.M.); 2 Urology Department, Norfolk and Norwich University Hospital, Colney Lane Norwich NR4 7UY, UK; robert.mills@nnuh.nhs.uk; 3 The Liggins Institute, University of Auckland, 84 Park Road, Grafton, Auckland 92019, New Zealand

**Keywords:** nutrition, bioactives, phytochemicals, prostate cancer

## Abstract

Prostate cancer has become the most common form of non-cutaneous (internal) malignancy in men, accounting for 26% of all new male visceral cancer cases in the UK. The aetiology and pathogenesis of prostate cancer are not understood, but given the age-adjusted geographical variations in prostate cancer incidence quoted in epidemiological studies, there is increasing interest in nutrition as a relevant factor. In particular, foods rich in phytochemicals have been proposed to reduce the risk of prostate cancer. Epidemiological studies have reported evidence that plant-based foods including cruciferous vegetables, garlic, tomatoes, pomegranate and green tea are associated with a significant reduction in the progression of prostate cancer. However, while there is well-documented mechanistic evidence at a cellular level of the manner by which individual dietary components may reduce the risk of prostate cancer or its progression, evidence from intervention studies is limited. Moreover, clinical trials investigating the link between the dietary bioactives found in these foods and prostate cancer have reported varied conclusions. Herein, we review the plant bioactives for which there is substantial evidence from epidemiological and human intervention studies. The aim of this review is to provide important insights into how particular plant bioactives (e.g., sulfur-containing compounds, carotenoids and polyphenols) present in commonly consumed food groups may influence the development and progression of prostate cancer.

## 1. Introduction

The intimate relationship between the physical and chemical structure of individual foods, dietary patterns and human health is complex. Diets high in saturated fats have been found to be associated with an increased risk of type 2 diabetes, cardiovascular disease (CVD) and cancer [1], whilst diets rich in fruit and vegetables may reduce blood pressure and lower the risk of developing chronic diseases. Cancer is defined as an uncontrollable proliferation of cells, and is the second leading cause of death worldwide after CVD [2]. More than half of all cancer diagnoses are considered preventable [3]. The International Agency for Research on Cancer (IARC) estimates the global burden of cancer to have risen to 18.1 million new cases and 9.6 million deaths in 2018 [4].

As a result of prostate-specific antigen (PSA) testing and an ageing population, prostate cancer (PCa) has become the most commonly diagnosed form of non-cutaneous (internal) cancer in men, accounting for 26% of all new male visceral cancer cases in the UK, with over 47,000 new diagnoses every year [5]. PCa incidence is strongly related to age, with more than a third (35%) of new cases occurring in males aged 75 and over [5,6]. 

The highest age-standardised incidences of PCa worldwide are seen in Westernised countries, including Australia, New Zealand, Europe and the Americas, compared to the lowest in South Central Asia [7,8]. This geographical variation in incidence cannot be explained by known risk factors such as age, race and family history [6], and implicates environmental factors, including diet [8,9,10], in the risk of developing PCa. For example, the incidence of PCa in Asian countries is low compared to the West, but this incidence rapidly increases (20-fold increased risk) in Asian immigrants to the United States that have adopted a Western diet, reducing intakes of soy, tea, fish, fruits, and vegetables and increasing their intake of red meat and fat-rich food [6]. Indeed, the recent rise in incidence and mortality of PCa in Eastern countries (although this may in part reflect rising capacity for diagnosis as opposed to true incidence) could partly be explained by the transition to a more Westernised lifestyle [8]. In addition, a study conducted in Western Australia has reported that the incidence of aggressive PCa is 80% higher in those consuming a Western diet in comparison to those consuming a high vegetable or “health conscious” diet [11]. The difference in PCa rates thus implicates dietary, lifestyle, and environmental factors in the development and progression of PCa [8,10,12]. 

Many epidemiological and case–control studies suggest that dietary factors such as animal fat, dairy products and red meat increase the risk of PCa, whereas the intake of fruit and vegetables is protective [13,14,15]. A Mediterranean diet with high intake of fish, legumes and olive oil has also been linked with a reduced PCa risk [15,16], and bioactive compounds such as sulfur metabolites found in cruciferous and alliaceous vegetables, epigallocatechin gallate in green tea, lycopene in tomato, curcumin in turmeric and polyphenols present in pomegranate and red wine have all received interest as possible protective agents in the diet [3,17,18,19,20]. A double-blinded placebo-controlled randomised trial has shown that patients with localised PCa on active surveillance who consumed a whole-food supplement containing a combination of broccoli powder, turmeric powder, pomegranate whole fruit powder and green tea extract for 6 months had a significantly lower median PSA rise (14.7%; 95% CI: 3.4%–36.7%) when compared to a placebo control (78.5%; 95% CI: 48.1%–115.5%) (*p* = 0.0008) [21], with PSA being one of the few biomarkers associated with risk of PCa. This study highlights the potential inverse association of multiple plant bioactives within the diet on PCa progression, although the role that each compound individually may contribute is unclear.

The occurrence of cancer in the prostate is often associated with the development of what appear to be several apparently independent cancer foci. Recent studies have also shown that histologically “healthy” prostate tissue in ageing glands have many mutations that are often associated with carcinogenesis, which may facilitate the emergence and proliferation of cancerous clones [22]. Diet and particularly plant bioactives may be able to directly or indirectly modify the cellular environment of the prostate and inhibit the emergence of new cancer clones, in addition to preventing the proliferation of existing clones [23].

Several reviews have been conducted highlighting the potential impact of plant-derived bioactives and PCa [17,18,19,20,24]. This review will focus on recent evidence from epidemiological and human intervention trials for the PCa-preventative properties of three classes of plant-derived bioactives for which there is substantial evidence: sulfur-containing metabolites from cruciferous and alliaceous vegetables (glucoraphanin, sulforaphane, S-alk(en)yl-L-cysteine sulfoxides and S-methyl-L-cysteine sulfoxide), carotenoids (lycopene) and polyphenols (resveratrol, catechins, curcumin and elligatannins). Moreover, in contrast to previous reviews, this review also considers pre-clinical research studies in order to gain further indications of potential mechanisms by which individual foods and food components may reduce risk of PCa.

## 2. Sulfur-Containing Bioactives from Cruciferous and Alliaceous Vegetables and Prostate Cancer

### 2.1. Glucoraphanin and Sulforaphane from Cruciferous Vegetables

Cruciferous vegetables, including broccoli, Brussels sprouts and cauliflower, accumulate sulfur-containing glycosides known as glucoisnolates (GSLs), which contribute to the organoleptic properties of these vegetables. GSLs do not exhibit biological effects themselves but are enzymatically hydrolysed either by the plant enzyme myrosinase or by bacterial myrosinases present in the gut microbiota, giving rise to breakdown derivates including indoles, thiocyanates and isothiocyanates [25].

**Figure 1:**
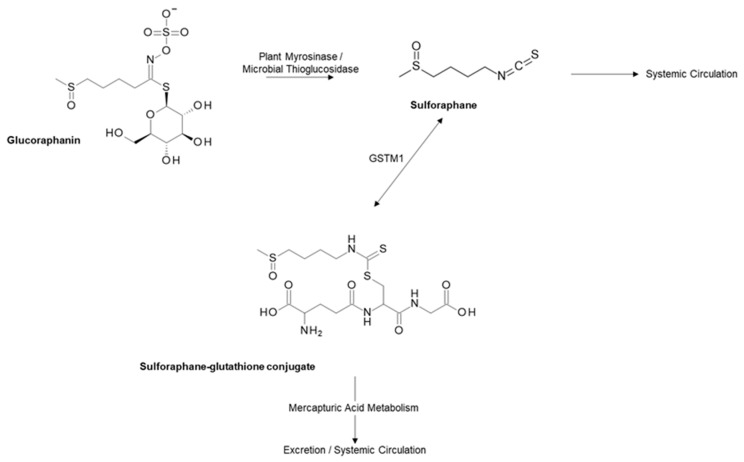
Metabolism of glucoraphanin. Sulforaphane is absorbed readily into the enterocyte and conjugated with glutathione via the GSTM1 enzyme. Sulforaphane is then either metabolised and excreted in the urine via the mercapturic acid pathway or cleaved from glutathione into free sulforaphane [25].

The strongest evidence with regards to a reduction in PCa incidence and progression is for the 4-methylsulfinylbutyl glucosinolate glucoraphanin (GRA), the most abundant glucosinolate found in broccoli. GRA is converted to the isothiocyanate sulforaphane (SFN) by the plant enzyme myrosinase, or in the gut by bacteria with microbial thioglucosidase activity (Figure 1) [24].

Briefly, when GRN has been hydrolysed into SFN, it is passively absorbed into the enterocyte of the small or large intestine, where it is conjugated (either passively or via the activity of glutathione-S-transferases (GSTs)) with glutathione, for transportation into the systemic circulation. The conjugate is then either metabolised via the mercapturic acid pathway to produce thiol conjugates which are excreted in the urine, or dissociates or is cleaved by GST, to become free SFN (Figure 1) [25,26].

SFN has been shown to have multi-modal influences on a wide variety of metabolic and cell-signalling pathways involved in cancer including the induction of antioxidant pathways, induction of apoptosis in cancer cells, inhibition of inflammation and angiogenesis and detoxification of carcinogens [27]. The most widely researched molecular target of SFN in relation to the induction of an antioxidant response is nuclear factor erythroid 2-related factor 2 (Nrf-2) [28]. Nrf-2, a transcription factor and regulator of nuclear factor-kappa B (NF-κB), is bound in the cytosol as a complex with Kelch-like ECH-associated protein (Keap1), which negatively regulates its transactivation potential. When reactive oxygen species are present (and indeed in the presence of SFN), the Nrf-2:Keap1 complex dissociates which enables the translocation of Nrf-2 to the nucleus, whereby it can exhibit its effects and induce detoxification genes [29]. Through this mechanism, SFN has been shown to regulate phase I and II detoxification enzymes, nicotinamide adenine dinucleotide phosphate (NADPH) regeneration and antioxidant defence [26]. SFN may also be able to directly bind and inhibit NF-κB (known to be elevated in cancer) and subsequently reduce tumour cell proliferation [30,31].

Research suggests that SFN also plays a significant role in the metabolic regulation of the prostate, thus leading to decreased PCa incidence and progression [32,33]. Previously, SFN has been shown to inhibit the occurrence of prostatic intraepithelial neoplasia in TRAMP (transgenic adenocarcinoma of the mouse prostate) mice through increased cytotoxicity of natural killer cells [32]. However, more recently the treatment of LNCaP and castration-resistant (22Rv1) human PCa cells with SFN has shown to downregulate fatty acid (FA) metabolism proteins, including those involved in FA synthesis (acetyl-CoA carboxylase 1 (ACC1) and fatty acid synthase (FASN)) and FA uptake for β-oxidation (carnitine palmitoyltransferase 1A, CPT1A). Furthermore, SFN-treated TRAMP mice demonstrate a significant reduction in plasma and prostatic adenocarcinoma levels of free FAs, suggesting that the reduction in PCa progression by SFN is linked with reduced FA metabolism [33]. 

Alterations in the acetylation patterns of histones are a characteristic feature in PCa progression [34]. SFN has been shown to interfere with a key androgen receptor (AR) chaperone, Hsp90, by inhibition of histone deacetylase (HDAC) enzymes, which remove acetyl groups from histones, inhibiting HDAC activity within cancer cell lines [35]. SFN has been shown to directly attenuate the AR pathways present in the LNCaP PCa cell line via the inhibition of HDAC6 activity [36]. In addition, SFN has been shown to inhibit epigenetic regulators including HDAC3 in PC3 PCa cell lines and decrease protein expression in the TRAMP model [37]. SFN metabolites (sulforaphane-cysteine (SFN-Cys) and sulforaphane-N-acetyl-cysteine (SFN-NAC)) have been shown to induce the phosphorylation of extracellular signal-regulated protein kinases 1 and 2 (ERK1/2), leading to microtubule disruption and apoptosis in DU145 and PC3 human PCa cell lines [38]. 

Consistent with cell and animal models, epidemiological studies have demonstrated a link between the intake of cruciferous vegetables (particularly the active compound SFN) and a reduction in the incidence or progression of PCa [39,40,41,42,43]. A large data set meta-analysis including six population-based case–control studies and seven cohort studies showed a significantly decreased PCa risk overall for cruciferous vegetable intake (RR = 0.90; 95% CI: 0.85–0.96) as well as in the subgroup of case–control studies (RR = 0.79; 95% CI: 0.69–0.89) [39]. In addition, a prospective study involving patients with extra-prostatic disease documented a 59% reduced risk of PCa progression for highest vs. lowest intake of cruciferous vegetables (HR: 0.41; 95% CI: 0.22–0.76, *p* = 0.003) [40]. However, there are few human intervention studies which support these findings.

In one such randomised control trial (RCT) involving 98 patients scheduled for a prostate biopsy as part of their routine clinical care (who had not previously received any PCa-related treatment procedures), patients consumed either capsules containing myrosinase-treated broccoli seed extract (BSE) containing 100 µmol of SFN twice daily, or a matched placebo control for 4–6 weeks prior to their biopsy procedure [44]. This study demonstrated a significant accumulation of total SFN and individual SFN metabolites in both the urine and plasma when compared to the placebo control. In addition to these findings, gene expression analysis identified three significantly differentially expressed genes in the supplementation arm when compared to placebo. Despite this, however, a difference in levels of other epigenetic PCa biomarkers was not demonstrated.

A further human intervention trial undertaken in Norfolk (UK) has demonstrated that the several hundred changes in gene expression and potentially oncogenic pathways (which are consistent with increased risk of carcinogenesis in normal prostate tissue) are suppressed in a dose-dependent manner by ingesting broccoli soups with increasing concentrations of GRN [23]. This three-arm parallel randomised double-blinded interventional study was conducted over a 12-month period, involving 49 participants on “active surveillance” for PCa. Participants received a weekly 300 mL portion of soup made from either a standard broccoli (control) or from one of two experimental broccoli genotypes with enhanced concentrations of GRN for a period of 12-months, prior to a template-guided transperineal prostate biopsy (TPB). The broccoli genotypes used included a commercially available control broccoli, a commercial variety (Beneforté™) delivering 3× the GRN level to that of the control [45] and a non-commercial variety delivering 7× the GRN of the control. These results provide a mechanistic explanation of how consumption of the cruciferous vegetable broccoli may lead to a reduction in the risk of PCa progression.

### 2.2. S-Alk(en)yl-L-Cysteine Sulfoxides (SACSOs) from Alliaceous Vegetables

**Figure 2:**
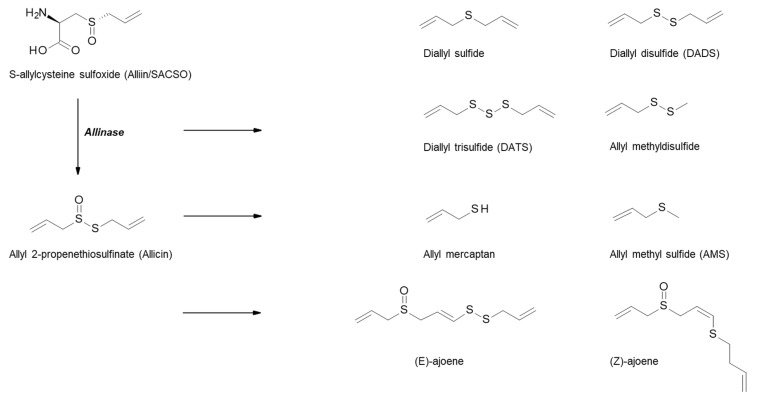
Metabolism of organosulfur compounds in garlic. Schematic outlining the breakdown of the S-alk(en)yl-L-cysteine sulfoxide (SACSO) alliin initially by alliinase, to thiosulfinates, and further compounds including sulfides [38,39,46].

Alliaceous vegetables, including garlic, onions, leeks and shallots, are rich in a variety of bioactive compounds, such as flavonols, oligosaccharides, selenium, arginine and organosulfur compounds (OSCs), the latter being of most interest with regards to health benefits, especially in relation to cancer. The OSCs within *Allium* vegetables are responsible for their characteristic odour and flavour and comprise approximately 1% dry weight of garlic and 0.5% dry weight of onions. The S-alk(en)yl-L-cysteine sulfoxides (SACSOs) are the main sulfur-containing constituents within allium vegetables, and are precursors to the bioactive components. Alliin (S-allylcysteine sulfoxide) is the major SACSO found in garlic, and isoalliin (*trans*-(+)-S-(propen-1-yl)-L-cysteine sulfoxide) is the predominant SACSO in onions [37,38,39]. When garlic is cut, chopped, or crushed, the disruption of the cell membranes causes the transformation of SACSOs to sulfenic acid intermediates by the enzyme alliinase, which is released from the vacuoles of the plant as part of its defence system. These intermediates are highly reactive and rapidly produce thiosulfinate compounds via condensation reactions (Figure 2). Allicin (thio-2-propene-1-sulfinic acid S-allyl ester) is the major garlic thiosulfinate, and due to its unstable nature, is rapidly broken down to further compounds such as ajoene, vinyldithiins, and sulfides including diallyl sulfide (DAS), diallyl disulfide (DADS), and diallyl trisulfide (DATS) [46].

Multiple cell and animal models have suggested a protective role of alliaceous vegetables in cancer risk, with several reported mechanisms, each targeting most of the cancer hallmarks defined by Hanahan and Weinberg [47], such as caspase-dependent and independent apoptosis induction, detoxification of carcinogens and cell cycle arrest [48,49,50]. Some components of alliaceous vegetables are reported to block the metabolism of hydrocarbons and nitrosamines and modulate phase I and II enzymes and DNA repair [51]. In addition, a further study has shown that S-allylmercaptocysteine can modulate the expression of androgen-responsive biomarkers and hence alter the androgenic action in prostatic cells [52].

Large-data-set epidemiological studies have supported this suggested negative correlation between the intake of alliaceous vegetables and the incidence of PCa [53,54]. Results from a large Shanghai-based case–control study showed that men ingesting more than 10 g/day of allium vegetables had a reduced risk of PCa compared with those who consumed less than 2.2 g/day (OR = 0.51; 95% CI: 0.34–0.76, *p* < 0.001), with the effect being most pronounced for garlic and scallions, and for the risk of developing localised PCa [53]. Research has shown that garlic consumption in the Shanghai population is significantly higher than in British population studies (46% of Shanghai males consume at least 6 g (approx. two cloves) of garlic a week compared to 15% of British males), compared to a higher consumption of onions in Western populations [55]. A further systematic literature review of nine studies across four continents (including China, the Netherlands, Italy and the USA) reported a significantly decreased risk of PCa with increasing intake of alliums overall (OR = 0.80; 95% CI: 0.70–0.92), and in particular for garlic (OR = 0.77; 95% CI: 0.64–0.91), but not for onions [54]. 

There are currently no published human intervention studies analysing the effect of alliaceous vegetables on PCa. As part of a recently commenced randomised double-blinded clinical trial in Norwich (UK) (clinicaltrials.gov code NCT04046653) patients will consume dietary supplements of SACSO for 4–6 weeks prior to a TPB. This study aims to assess whether SACSO or its metabolites accumulate within the urine, plasma or prostate tissue, as well as modify the gene expression, when compared to a placebo control. This trial will contribute to our understanding of how these bioactives influence PCa progression. 

### 2.3. S-Methyl-L-cysteine Sulfoxide (SMCSO) from Cruciferous and Alliaceous Vegetables

**Figure 3:**
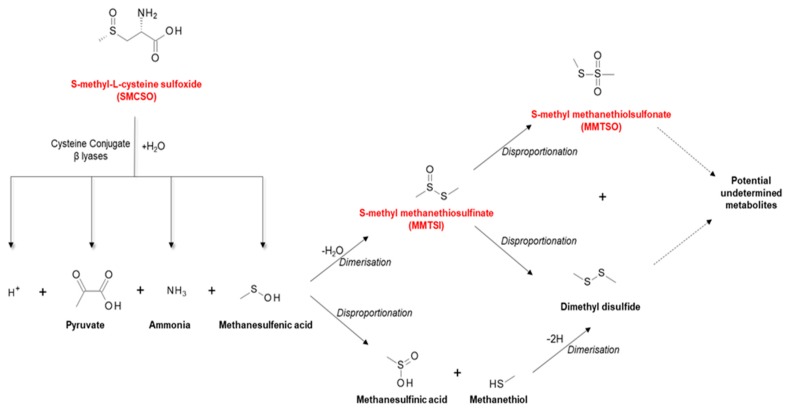
S-methyl-L-cysteine sulfoxide (SMCSO) metabolism. Schematic outlining the breakdown of SMCSO initially by specific cysteine conjugate β lyases leading to secondary bioactive products through dimerization and disproportionation reactions. Highlighted in red are the sulfur-containing metabolites with potential undetermined biological activity [57].

In addition to glucosinolates, cruciferous vegetables including broccoli contain other sulfur-containing metabolites, such as S-methyl-L-cysteine sulfoxide (SMCSO; methiin). SMCSO is found in higher concentrations (dry weight of 1%–4%) in comparison to glucosinolate concentration (0.1%–0.6%) [48]. SMCSO is metabolised by plant- or microbial-based specific cysteine conjugate β lyases, thus generating the biologically relevant volatile sulfur compounds S-methyl methanethiosulfinate (MMTSI) and S-methyl methanethiolsulfonate (MMTSO) [56]. MMTSO is produced through disproportionation of MMTSI (pathway shown in Figure 3). The full mechanism for SMCSO metabolism is yet to be fully elucidated, but the gut microbiota appears to play a significant role [57].

In vitro studies on the effect of SMCSO and its breakdown products MMTSI and MMTSO on human PCa cells lines are well documented [56], but there have been limited human clinical interventional studies investigating this relationship. To date, the pathway of SMCSO in humans has been described twice. Following fourteen days of the administration of radiolabelled SMCSO and S-carboxymethyl-L-cysteine sulfoxide (SCMCSO) to four healthy males, the radiolabelled compounds had completely degraded to sulfate, with urine being the major pathway of excretion (96% in 14 days) [58]. More recently, SMCSO has been shown to accumulate within the prostate and peri-prostatic tissue of men consuming a high-GRN broccoli soup three times a week for 4 weeks prior to a TPB. SMCSO was also detected within urine, and its levels were correlated with those in prostate tissue [57]. There are no known routes by which mammals can synthesise SMCSO, which makes a dietary origin most likely.

Along with the S-alk(en)yl cysteine sulfoxides, SMCSO is also known to accumulate within alliaceous vegetables such as garlic. As described above, SMCSO has been shown to accumulate within the prostate and urinary system [57]. This compound is structurally very similar to SACSO, which supports the theory that SACSO and its metabolites may accumulate within the prostate or urinary system when consumed at high concentrations, and may therefore be responsible for beneficial health effects. The role of SMCSO in PCa prevention is yet to be elucidated, but is an area of great interest.

## 3. Carotenoids and Prostate Cancer

### Lycopene from Tomatoes

**Figure 4:**
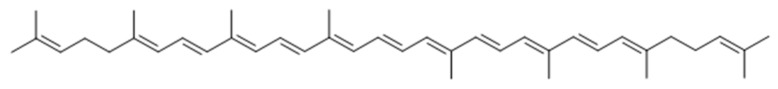
Chemical structure of lycopene.

Lycopene is the most abundant carotenoid found in tomatoes, and accounts for >85% of all dietary sources [59]. It is a 40-carbon atom, acyclic open-chain hydrocarbon containing 11 conjugated and 2 non-conjugated double bonds, assigned in linear array building a chromatophore (Figure 4). These double bonds allow for potential extensive isomerisation, which could theoretically result in 1056 *cis–trans* configurations [60]. Despite this, there are only a few isomers found in nature, the most prevalent being the all-*trans*-isomer (the all-*trans* and 5-*cis* isomers being the most thermodynamically stable configurations). Isomerisation from *trans*-isomer to various other forms can occur secondary to heat, light, and chemical reactions or secondary to in vivo mechanisms [61,62]. 

The lycopene concentration in fresh fruit varies greatly; up to 15 mg of lycopene has been found per 100 g of deep-red tomato varieties, compared with only 0.5 mg per 100 g of yellow tomatoes [62,63]. Processed tomato-based foods have been shown to contain higher levels of lycopene than fresh raw tomatoes, with tomato sauces containing >17 mg/100 g and tomato paste containing >55 mg/100 g [64]. The colour of the lycopene is directly related to its isomeric form, with all-*trans* isomers being deep red, compared with tetra-*cis*-lycopenes which are more orange in appearance [65].

**Figure 5:**
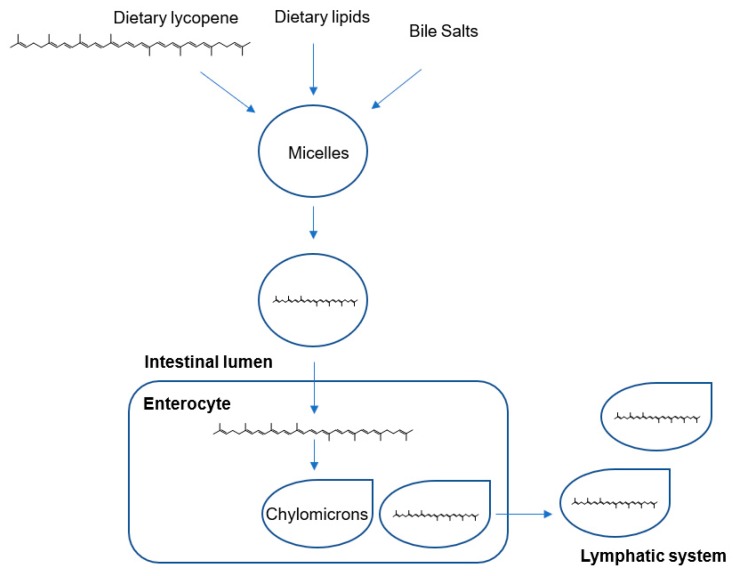
Digestion and absorption of lycopene in the small intestine. Lycopene is absorbed into the enterocyte in lipid micelles and transported in the lymph in chylomicrons to the liver prior to transportation in plasma to target organs [65].

Lycopene is passively absorbed in the small intestinal mucosa via integration into dietary lipid micelles, along with other lipids and bile acids. Micelles are transported from the enterocyte to the liver in the form of chylomicrons in lymphatic fluid, prior to transportation in the plasma to target organs via low-density and very-low-density lipoproteins (Figure 5). The absorption of lycopene ranges between 10%–30% and is dependent upon many factors, including age, smoking, alcohol consumption and dietary composition [66,67]. Importantly, the lipid-soluble nature of lycopene means that its bioavailability is vastly increased by consuming it with fat [68]. Lycopene appears to distribute unevenly across organs, perhaps due to differing metabolic/oxidation rates or lipoprotein receptor numbers [69,70], however, lycopene preferentially accumulates to maximal concentrations in the testes, adrenal glands, liver and prostate [66,70].

The multiple conjugated double bonds are believed to be responsible for lycopene’s various protective (anti-oxidant) effects. Its active singlet oxygen (free radical) quencher is believed to assist DNA repair and may react with oxygen free radicals by transfer of the unpaired electron, leaving the carotenoid in an excited triplet state. Indeed, lycopene has been shown to exhibit a singlet quenching ability twice as powerful as beta-carotene and tenfold greater than alpha-tocopherol (vitamin E) [71].

Considerable in vivo work has demonstrated a potential antiproliferative effect of lycopene on PCa cells, with postulated mechanisms linked to cell cycle arrest and apoptosis. Lycopene has been shown to exert antiproliferative effects via its ability to induce G2/M-phase cell cycle arrest and apoptotic cell death on LNCaP cells, especially in unsynchronised cells [72]. These findings have been reproduced by further studies [73]. Similarly, the treatment of androgen-independent PCa DU145 cells with lycopene has shown to induce G0/G1-phase cell cycle arrest and induction of apoptosis in a dose-dependent manner (apoptosis rate increased by 42.4% in cells treated with 32 µmol/L lycopene compared to controls) [74]. Features of apoptosis induction such as reduced mitochondrial potential, release of mitochondrial cytochrome c and annexin V binding have been shown to upregulate when LNCaP cells are treated with increasing concentrations of lycopene [75]. A recently published systematic review of cell and animal studies also suggests that lycopenes have the ability to interact with the androgen axis, downregulating androgen metabolism and signalling in PCa [76]. 

Most of the data linking the association between lycopenes and PCa originate from epidemiological or prospective studies. The literature surrounding the association with lycopene and PCa is mixed and remains controversial, particularly with reference to aggressive and/or advanced PCa. A systematic review and meta-analysis of 42 case–control studies (43,851 PCa cases) published up to 2016 showed that dietary intake (RR = 0.88; 95% CI: 0.78−0.98, *p* = 0.017) and circulating concentrations (RR = 0.88; 95% CI: 0.79−0.98, *p* = 0.019) of lycopene were associated with reduced (localised) PCa risk [77]. This association was also shown to be dose-dependent; for every additional 2 mg of lycopene, the risk of PCa decreased by 1% (*p* = 0.026), and PCa risk decreased by 3.5% to 3.6% for each additional 10 μg dL^−1^ of circulating lycopene. However, this association was not shown to be protective against the incidence of advanced PCa. Similarly, a systematic review and dose–response meta-analysis of dietary intake of lycopenes in relation to PCa risk also showed a dose-dependent associated reduced risk of PCa incidence (RR for dietary lycopene intake: 0.86, 95% CI: 0.75–0.98; RR for blood lycopene levels: 0.81, 95% CI: 0.69–0.96), with no protection to the risk of advanced PCa [78]. Dose–response analysis demonstrated a reduced risk of PCa by 3% per 1 mg/day (95% CI: 0.94–0.99) increment of dietary lycopene intake.

A further case–control study of 408 controls in a Vietnamese population showed that higher intakes of total carotenoids were significantly associated with a reduced risk of a PCa diagnosis. Of all carotenoid intakes, lycopene exhibited the most significant results, conferring an adjusted OR of 0.46 (95% CI: 0.27–0.77) of PCa risk when comparing the highest versus lowest tertile of lycopene intake [79]. These inverse associations were dose-responsive and independent of other confounding factors commonly associated with PCa, including age, family history of PCa and body mass index.

In contrast to the above studies, a pooled analysis of 15 studies failed to show any association between the intake of seven major carotenoids and overall risk of PCa. However, high lycopene intake was shown to be inversely associated with risk of advanced stage PCa and aggressive disease (OR 0.65; 95% CI: 0.46–0.91, *p* = 0.032) [80].

As is true for all nutrients, estimation of dietary lycopene intake through the use of questionnaires often creates bias [81]. Further bias is introduced via bioavailable lycopene measurement error. A review of 11 studies comparing dietary lycopene intake with circulating levels document an average correlation of 0.2 [82]. However, in one prospective study, participants consuming a high intake of tomato sauce were found to have the highest published correlation of dietary and circulating lycopene (r = 0.46), indicating that the consumption of tomato sauce captures most of the bioavailable lycopene in the diet [82]. In this study, lycopene intake was associated with a reduced risk of PCa (RR = 0.84; CI: 0.73–0.96, *p* = 0.03) for high versus low quintiles, with the greatest risk reduction attributed to high tomato sauce intake (RR = 0.77; 95% CI: 0.66–0.90, *p* < 0.001 for >2 servings/week vs. <1 serving/month) especially for extra-prostatic cancers (RR = 0.65; 95% CI: 0.42–0.99). In addition to highly concentrated sources of carotenoids found in tomato sauces, the thermal processing involved in the cooking of tomato sauces acts to increase the bioavailability of lycopene; heat causes the binding matrices to disrupt, and an oily base improves uptake into micelles and subsequent absorption in the intestine [82].

These findings are supported by the results of a clinical study in which 32 patients with biopsy-confirmed PCa were randomised into either a treatment arm (consuming 30 mg lycopene/day in the form of a tomato sauce) or a control arm, for 3 weeks prior to prostatectomy. Intervention with a tomato sauce resulted in an increased abundance of apoptotic cells (from 0.84% ± 0.13% to 2.76% ± 0.58%; *p* = 0.0003) and degree of apoptotic cell death (from 0.84% ± 0.13% to 1.17% ± 0.19%; *p* = 0.028) in resected tumour areas when compared to the non-intervention controls [83]. 

However, results from the post-hoc analysis of the “Procomb trial” in which patients were followed up for two years following dietary intervention (and underwent a prostate biopsy when there was either a clinical suspicion of PCa or PSA rise to >4 ng/mL) did not provide any evidence for lycopene supplementation in terms of protection from PCa incidence. Supplementation in this trial also included selenium. Larger human studies are required to elucidate the potential anti-PCa effects of lycopene [84].

## 4. Polyphenols and Prostate Cancer

### 4.1. Resveratrol from Wine

**Figure 6:**
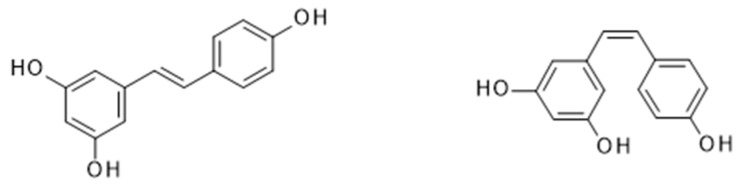
Isomeric forms of resveratrol: *trans-* (**left**) and *cis-* (**right**).

Resveratrol (*trans*-3,5,4’-trihydroxystilbene) is a stilbenoid polyphenol belonging to the phytoestrogens. Two isomeric forms of resveratrol exist; the *trans-* isomer is the most stable and biologically active form, whereas the *cis*- isomer is formed following the breakdown of the *trans-* form secondary to the action of UV light or high-pH conditions during fermentation (Figure 6). Both isomeric forms of resveratrol can be found in variable concentrations in grapes, as well as several fruits including tomatoes, raspberries, blueberries and mulberries. The highest concentration of resveratrol is found in the skin of black and red grapes (50–100 µg/g), and thus, red wine, and to a lesser degree in white and rosé wines. Commercially available wines have been shown to contain a concentration of resveratrol in the range of 0.1–14.3 mg/L [85,86], with the Pinot Noir grape varieties containing the highest concentrations of *trans*-3,5,4′-trihydroxystilbene [87]. Resveratrol is also found in cocoa and chocolate to a lesser degree (1.85 ± 0.43 µg/g and 0.35 ± 0.08 µg/g, respectively) [85,86,88].

Following ingestion, resveratrol is passively absorbed via transepithelial diffusion into the small intestine, where it undergoes immediate and extensive intestinal and hepatic metabolism (via conjugation with glucuronic acid). Oral absorption of resveratrol is documented as being as high as 75%. Following metabolism, the major metabolites occurring in plasma and urine are sulfates and glucuronides (via deconjugation enzymes such as B-glucuronidase), reduced conjugates of which can accumulate to varying concentrations in tissue. Although intake of 5 g resveratrol daily is considered to be safe, evidence suggests that a daily intake of 0.5–1 g can elicit can reduce tumour cell proliferation in the GI tract [89], with mild to moderate gastrointestinal symptoms occurring at doses of 2.5–5 g [85,90], and other mild side effects such as headaches occurring at higher doses [91].

Resveratrol appears to have a significant multi-modal anti-tumoral activity on PCa cells via increasing apoptosis, cell cycle arrest and downregulation of signal transduction pathways. Resveratrol has been shown to have proapoptotic effects on multiple PCa cell lines, including LNCaP, DU–145 and PC–3; and in the athymic xenograft LNCaP and TRAMP mouse models [85]. In addition, resveratrol increases the proapoptotic potential of TNF-related apoptosis inducing ligand (TRAIL) by activating the FOXO family of forkhead transcription factors (e.g., FKHRL1) and its target genes [92]. TRAIL, a cytokine which incurs a low toxicity to non-malignant cells, has been shown to induce apoptosis in 60% of multiple PCa cell lines. The metastatic potential of PCa cells is also altered under the influence of resveratrol, via the reduction in expression of vascular endothelial growth factor (VEGF) and VEGF receptor 2 (VEGFR2), including the matrix metalloproteinases (MMPs) [92]. In addition, both resveratrol and its analogues have been shown to regulate to action of metastasis-associated protein 1 (MTA1) and microRNA [93]. Furthermore, resveratrol has demonstrated a direct and potent inhibitory effect (even at low concentrations) on DU145 PCa cells, as measured by the reduction of reactive oxygen species (ROS) production [94].

Resveratrol has also been shown to down-regulate AR expression in TRAMP mouse models [95], and alter the AR and chemokine receptor type 4 (CXCR4) pathways required for tumour progression and metastasis [96]. CXCR4, a chemokine receptor which is upregulated in cancer metastasis, is significantly inhibited by resveratrol (both alone and in combination with bicalutamide), in addition to a reduction in the downstream genes associated with cell cycle progression. Importantly, resveratrol also appears to act on androgen-independent cells. In the presence of resveratrol, mediators of survival pathways such as phosphoinositide 3-kinase (PI3K) have been shown to decrease, leading to a reduction in the phosphorylation of downstream targets such as protein kinase B (PKB/Akt) [97].

Much of the clinical data supporting this relationship is derived from epidemiological studies. Alcohol intake has previously been associated with a significant dose–response increase in risk of PCa incidence and progression [98], but the type of alcohol consumption was not analysed. A recent meta-analysis of 17 studies (non-randomised observational or case–control studies) including over 600,000 subjects reported that “moderate” wine consumption did not increase the risk of PCa (0.98; 95% CI: 0.92–1.05, *p* = 0.57) [99]. The definition of “moderate” consumption differs across all included studies, although the maximum intake for all studies was one glass of wine per day. However, multi-variant analysis produced varied and antagonistic results depending upon wine type; the overall risk of PCa incidence increased with moderate intake of white wine (RR 1.26; 95% CI: 1.10–1.43, *p* = 0.001), whereas moderate red wine consumption demonstrated a protective role, with a risk reduction of 12% (RR 0.88; 95% CI: 0.78–0.999, *p* = 0.047). However, the exclusion of one large prospective study with a 16-year follow-up demonstrated no significant association between wine type and risk of PCa [100]. However, epidemiological studies involving alcohol are often prone to significant reporter bias. Additionally, it remains difficult to separate the potential beneficial effects of resveratrol from the known and detrimental effects of ethanol [98].

A randomised placebo-controlled clinical study undertaken in middle-aged men with metabolic syndrome has shown that high-dose dietary supplementation of resveratrol for 4 months (1000 mg/day) resulted in significantly lower serum levels of androgen precursors than a control. Androstenedione, dehydroepiandrosterone (DHEA) and dehydroepiandrosterone sulfate were all shown to be lowered by up to 50% (24% *p* = 0.052, 41% *p* ≤ 0.01 and 50% *p* < 0.001, respectively), although no effect on PSA, prostate size, testosterone or dihydrotestosterone levels was seen [101]. Future human studies are required to elucidate the clinical effect of resveratrol on PCa.

### 4.2. Catechins from Green Tea

**Figure 7:**
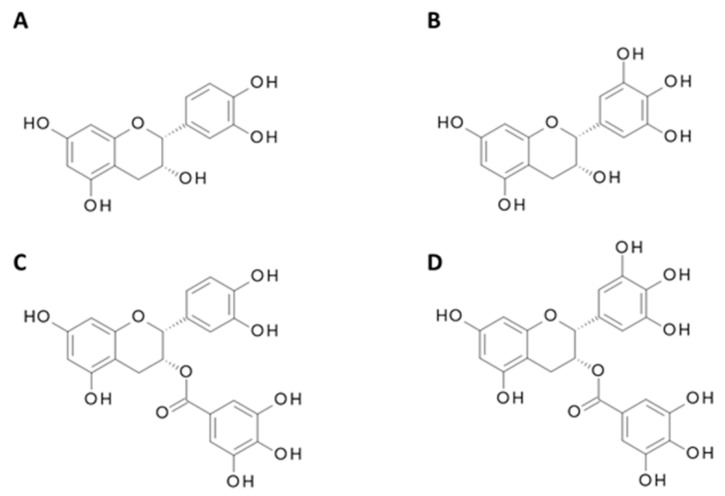
Chemical structure of major green tea catechins. (**A**) Epicatechin (EC); (**B**) Epigallocatechin (EGC); (**C**) Epicatechin 3-gallate (ECG); (**D**) Epigallocatechin-3-gallate (EGCG).

A variety of teas are produced from the leaves of *Camellia sinensis*, including green, black and oolong. The production of green tea differs from that of black tea and oolong in that it is made by steaming the fresh leaves, thus preventing the oxidation of the polyphenol compounds [102]. Green tea contains polyphenols known as catechins which include epicatechin (EC), epigallocatechin (EGC), epicatechin-3-gallate (ECG) and the most abundant epigallocatechin-3-gallate (EGCG), shown in Figure 7. Green tea catechins, specifically EGCG, have been heavily studied at an in vitro and epidemiological level giving vital insight into its effect on PCa incidence and progression. 

EGCG has been shown to be one of the most potent modulators of the molecular pathways involved in prostatic carcinogenesis [103,104]. Preclinical in vitro work on PCa cell lines exposed to EGCG have shown promising results. LNCaP PCa cells treated with 0–80 µM EGCG resulted in the suppression of cell proliferation (both dependent and independent of androgens) as well as a reduction in levels of PSA [105]. Treatment with 0–50 µM EGCG has been shown to prevent the proliferation of PC3 PCa cell lines with an IC_50_ value of 39 µM, via the activation of extracellular signal-regulated kinase (ERK1/2), independently of mitogen-activated protein kinase kinase (MEK) signalling [106]. Additionally, encapsulation of EGCG with polysaccharide nanoparticles induced apoptosis and reduced the cell viability of DU145 PCa cell lines more than free EGCG [107], suggesting a potential method for delivery that preserves the bioavailability of plant bioactives for chemoprevention.

EGCG influences other anti-cancer mechanisms through cell cycle arrest and apoptosis, including protein kinase C pathways, inflammatory pathways such as NF-_K_B cyclooxygenase-2 (COX-2), and the targeting of insulin-like growth factor (IGF) [108].

However, meta-analyses of epidemiological studies have reported conflicting results. Three meta-analyses have all demonstrated no significant inverse association between green tea intake and risk of PCa (RR = 0.75, 95% CI: 0.53–1.07 [109]; RR = 0.79, 95% CI: 0.43–1.14 [110]; and RR = 0.73, 95% CI: 0.52–1.02 [111]). However, following sensitivity analysis the most recent of these studies demonstrated significant heterogeneity caused by one individual study in which a Chinese population was consuming large amounts of green tea for >40 years [109,112]. Novel data excluding this study demonstrated that consumption of green tea catechins may reduce the risk of PCa by 4.5% (*p* = 0.08) for each cup of green tea consumed per day.

In the first placebo-controlled clinical trial undertaken analysing the effect of green tea on PCa incidence, 60 patients with high-grade prostatic intraepithelial neoplasia (HGPIN) were randomised into either a placebo group or intervention, receiving 600 mg of green tea catechins per day. Over a 12-month follow up period, the incidence of progression to PCa (as diagnosed by prostate biopsies) was significantly reduced in the treatment arm (RR 0.38; *p* = 0.02) [113]. However, these findings are contradicted by more recent studies, which demonstrate no reduction in risk of PCa in men with baseline HGPIN or atypical small acinar proliferation (ASAP) [114]. However, one RCT in which 93 patients with confirmed PCa consumed six cups of green tea, black tea or water (placebo) daily prior to prostatectomy demonstrated a significant uptake of green tea polyphenols in prostatic tissue, as well as significantly reduced levels of prostatic NF-κB in the green tea arm. In addition, participants consuming green tea also exhibited a systemic antioxidant effect (as measured by urinary 8-hydroxydeoxy-guanosine, 80HdG) [115].

Further human studies are required to investigate the effects of green tea on PCa incidence and progression, either alone or in combination with other treatment modalities.

### 4.3. Curcumin from Turmeric

**Figure 8:**
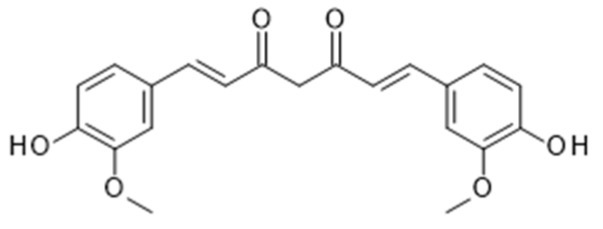
Chemical structure of curcumin.

Curcumin is a polyphenolic compound and secondary metabolite isolated from the rhizome of the plant *Curcuma longa* (commonly known as Turmeric), an herbaceous perennial plant of the ginger family (Figure 8). Curcumin has long been associated with health benefits, having been used for centuries in traditional Indian medicine for a variety of conditions [116]. Following ingestion, curcumin undergoes metabolic O-conjugation to curcumin glucuronide and curcumin sulfate, and bioreduction to additional metabolites including terahydrocurcumin, hexahydrocurcumin, octahydrocurcumin and hexahydrocurcumin. Reduced curcumin is also subjected to glucuronidation into curcumin glucuronide, dihydro-curcumin-glucuronide, tetrahydrocurcumin-glucuronide, and curcumin sulfate [117,118]. The majority of curcumin is metabolised in the liver and intestine, although small amounts remain detectable in blood, as well as in a variety of organs including the heart, lung, brain and kidney [118]. However, due to its poor absorption, extensive and rapid phase II metabolism, and rapid elimination, curcumin has a poor bioavailability. Several methods have been adopted to improve curcumin bioavailability, such as blocking these metabolic pathways. The known bioavailability enhancer piperine (the active component of black pepper) can increase curcumin bioavailability by 2000% by creating a curcumin complex, ultimately inhibiting hepatic and intestinal glucuronidation [119].

Like many dietary bioactives, the effect of curcumin on PCa cells is multi-modal. Curcumin has been proven in vitro to inhibit the growth of PCa cells by increasing apoptosis, suppressing NF-κB activation, downregulating the expression of B-cell lymphoma 2 (Bcl-2) and B cell lymphoma extra-large (Bcl-xL), and leading to an activation of caspase-3 and -8. Studies have confirmed the dose-dependent inhibition of PC3 cell viability, associated with a significant downregulation of mRNA and protein expression of inhibitor of DNA binding 1 (Id1) [120]. Daily intraperitoneal injections of curcumin in PC-3 xenograft mouse models support these findings with a significant reduction in tumour growth within a one-month period. Inhibitor of DNA binding proteins belong to a subgroup of helix-loop-helix (HLH) transcription factors, and are vital regulators of cell differentiation, and hence metastatic progression of cancers. Although Id1 is the most significantly researched, Id4 ectopic expression in DU145 PCa cells has also been shown to upregulate apoptosis, expression of p53 and halt cell proliferation secondary to S-phase arrest [121]. In addition, curcumin also appears to exhibit anti-carcinogenic effects via the activation of the Nrf-2 pathway [122]. Derivates of curcumin have been shown to enhance levels of Nrf-2 and phase II detoxifying genes through epigenetic regulation in TRAMP C1 PCa cells [123]. 

The increasing activity of the AR and the upregulation of AR-related co-factors such as cAMP response element-binding protein (CBP) and coactivator protein p300 are salient features of aggressive and hormone-resistant PCas. Curcumin has been shown to interact with these factors in LNCaP and PC-3 cell lines by inhibiting the expression of the AR [124], as well as suppressing occupancy at sites of AR function [125], thus reducing tumour growth and delaying the onset of hormone-resistant disease.

Multiple clinical trials have been undertaken to support these findings. However, most of the trials have described the use of curcumin as an adjunctive treatment to either radiotherapy, hormonal or chemotherapeutic interventions. A recently published RCT was undertaken in patients managed with intermittent androgen deprivation (IAD) for the treatment of biochemical recurrence of PCa following localised treatment or metastatic disease at diagnosis. Patients received curcuminoid powder in capsule form at a total dose of 1440 mg/day for 6 months, commencing at the time of androgen deprivation treatment (ADT) withdrawal. Although there was no significant difference in the overall “off-treatment” duration of IAD, curcumin treatment resulted in a significantly lower PSA progression in comparison to the control group (10.3% vs. 30.2%; *p*  =  0.0259) [126].

Due to the antioxidant, radiosensitising and radioprotective properties of curcumin, interest in the adjuvant treatment of curcumin with radiotherapy is gaining increasing interest. In a further RCT analysing oxidation status, patients received either adjuvant treatment of 3 g/day curcumin or placebo during external-beam radiotherapy (up to 74 Gy). Although plasma total antioxidant capacity (TAC) was seen to increase in all patients, this was significantly higher in the curcumin arm (*p* < 0.001), as was a reduction in the activity of superoxide dismutase (SOD) (*p* = 0.026). PSA, however, was shown to be reduced to <0.2 ng/mL in all patients, with no significant difference between treatment arms [127].

### 4.4. Ellagitannins from Pomegranate

**Figure 9:**
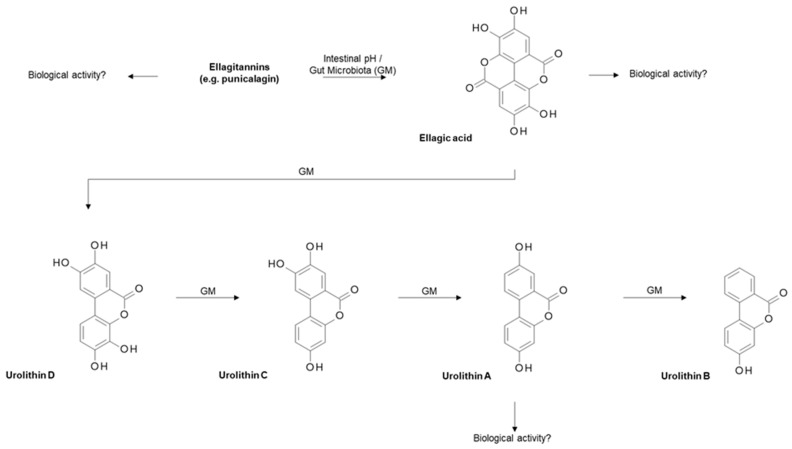
Metabolism of ellagitannin within pomegranate. Briefly, ellagitannins such as punicalagin are metabolised by intestinal pH and/or gut microbiota (GM) to give ellagic acid, which is further broken down by gut microbiota to give various urolithins, including urolithin A, which is biologically relevant [135].

Both the seeds and juice of the pomegranate fruit (from the tree *Punica granatum*) have been heavily researched with regards to their bioactive compounds, which are deemed to be anti-microbial, anti-inflammatory and anti-cancerous [128]. Pomegranate contains various beneficial components including high levels of vitamin C and polyphenols such as ellagitannins [129]. Ellagitannins, including punicalagin, are broken down following exposure to the intestinal pH and/or gut microbiota to ellagic acid, which is further metabolised by the gut microbiota to various urolithins, including the most biologically relevant, urolithin A (Figure 9).

Punicalagin has been shown to increase growth inhibition and apoptosis in two human PCa cell lines (PC3 and LNCaP), suggesting a potential anticancer activity of the bioactives within pomegranate [130]. Urolithin A has been shown to repress three PCa cell lines with differing p53 genotypes (LNCaP (p53 +/+), 22RV1(p53 −/+) and PC3 (p53 −/−), including an induction of apoptosis [131]. Tumour protein p53 is negatively regulated by the E3 ubiquitin-protein ligase MDM2 (mouse double minute 2 homolog) [132]. Urolithin A treatment led to increased p53 protein expression in 22RV1 and PC3 cells which subsequently resulted in the inhibition of MDM2-mediated p53 polyubiquitination [131], indicating that the presence of urolithin A possesses anti-cancer properties via its influence on the p53-MDM2 pathway. Pomegranate peel extract [133] and pomegranate leaves extract [134] have also been shown to upregulate apoptosis, reduce tumour cell proliferation and reduce the metastatic potential of TRAMP-C1, DU145 and PC3 PCa cells. 

In addition, pomegranate extract has been shown to exhibit an inhibitory effect on the NF-κB inflammatory pathway, thus enabling the extract to demonstrate its pro-apoptotic effect [136]. Increased NF-κB activity has been shown to be an important predictor of the biochemical recurrence of PCa and hence the transition from androgen dependence to independence following local therapy [136,137,138]. LAPC4 xenograft tumours (which exhibit a wild-type androgen receptor and features of hormone-dependent growth and metastasis [139]) treated with pomegranate demonstrate delayed regrowth following castration with a reduction in the increased levels of NF-κB activity [139,140]. Pomegranate extract has also been shown to be effective in treating androgen-independent PCa in mouse C4-2 xenografts with skeletal metastasis, both alone and in combination with docetaxel treatment [141]. In addition, the metabolites luteolin, ellagic acid and punicic acid have demonstrated PCa cell growth inhibition and reduced metastasis and angiogenesis in murine studies [142].

Much of the clinical research on pomegranate has focussed on its effects on CVD [143], but there has been limited and controversial clinical evidence analysing the influence of pomegranate fruit/juice on PCa. The first clinical trial of pomegranate juice in PCa patients was conducted in 2006, whereby 48 PCa patients consumed pomegranate juice daily for up to 33 months [144]. This phase II study demonstrated that those who drank pomegranate juice had a statistically significant elongation in PSA doubling time. These results were reproduced by a further clinical trial (*n* = 104) which demonstrated that the consumption of either 1 or 3 g pomegranate extract for up to 18 months resulted in an increase in PSA doubling time (from 11.9 months at baseline to 18.5 months after treatment; *p* < 0.001) [145]. However, in contrast, a short-term placebo-controlled trial (*n* = 102) concluded that daily pomegranate intake has no impact of levels of PSA in patients with advanced PCa at one month [146]. A further intervention study (*n* = 70) demonstrated that daily pomegranate supplementation for up to four weeks prior to radical prostatectomy resulted in urolithin A accumulation in prostate tissue (*p* = 0.031), but had no effect on prostatic oxidative stress, cell proliferation, tumour progression or PSA levels [147].

Whilst there is some evidence of pomegranate reducing PCa growth through murine studies and short-term intervention studies, the long-term impact of pomegranate consumption is not fully understood. Current clinical trials are ongoing and include: “Pomegranate Juice in Treating Patients with Recurrent PCa, ClinicalTrials.gov Identifier: NCT00060086” (data pending) which will contribute to our understanding of how these bioactives influence PCa progression. This study, in which up to 40 patients who have undergone radical prostatectomy or radiotherapy for PCa will consume oral pomegranate juice daily for 18 months, aims to determine whether pomegranate juice consumption can decrease or slow the rate of rising PSA levels. 

## 5. Exposure of Dietary Bioactives to the Prostate

**Figure 10:**
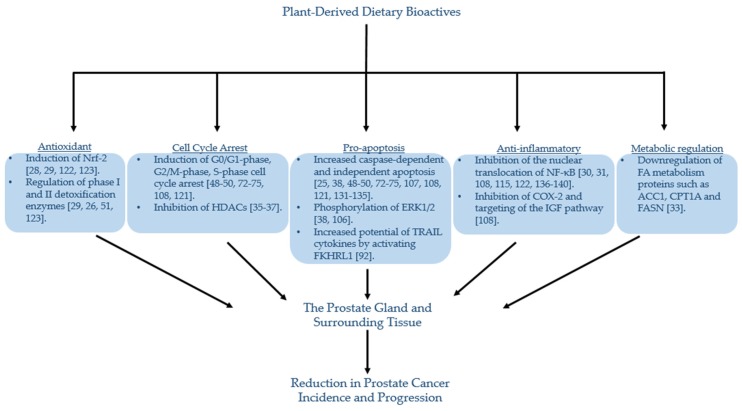
Potential mechanisms of dietary plant bioactives and the prevention of prostate cancer. Briefly, dietary plant bioactives have been associated with a reduction in prostate cancer incidence and progression through a variety of different mechanisms. These include antioxidant properties, pro-apoptosis, anti-inflammatory pathways, metabolic regulation and cell cycle arrest. Direct exposure of these bioactive compounds may occur secondary to urinary reflux via the urethral ductal system [154]. Abbreviations are as follows: ACC1: acetyl-CoA carboxylase 1; Bcl-2: B-cell lymphoma 2; Bcl-xL: B-cell lymphoma-extra-large; CPT1A: carnitine palmitoyltransferase 1A; COX-2: cyclooxygenase-2; ERK1/2: extracellular signal-regulated protein kinases 1 and 2; FA: fatty acid; FASN: fatty acid synthase; FKHRL1: FOXO transcription factor; HDAC: histone deacetylase; IGF: insulin-like growth factor; NF-κB: nuclear factor-kappa B; Nrf-2: nuclear factor erythroid 2-related factor 2; TRAIL: TNF-related apoptosis-inducing ligand.

From the evidence reviewed, it is clear that certain dietary bioactives may be associated with either a reduction in the risk and/or progression of PCa (Table 1). Studies in cell and animal model systems provide insight into a variety of mechanisms by which these bioactives may exert their effect (illustrated in Figure 10). However, despite well-documented associations, a clear understanding of the degree and mechanism of prostatic exposure to these dietary bioactives or their human or microbial metabolites is lacking. Exposure of the prostate is conventionally considered to be via the systemic circulation, within which these dietary bioactives would be at low concentrations compared to those used in in vitro studies, and extensively metabolised through phase 2 metabolism, (including sulfation, glucuronidation and methylation), which is likely to reduce their biological activity [148,149]. It is possible, however, that the prostate may be exposed to a higher concentration of these compounds though urinary reflux [150]. 

Evidence of the reflux of urine into the prostatic ducts was first published in 1982, when, following intra-vesical instillation of carbon microspheres, 70% of prostate tissue removed by transurethral resection was found to contain microspheres [151]. Additionally, granuloma formation following intra-vesical instillation of Bacillus Calmette–Guerin (BCG) used in the treatment of high-grade non-muscle-invasive bladder cancer occurs within the prostate as well as the primarily treated bladder [152,153].

Due to its ductal anatomy, the peripheral zone is the most likely part of the prostate to be exposed to urinary components. The ducts draining the peripheral glands enter the urethra as a double lateral line along the whole of the distal urethral segment. The ducts are narrow but enter the urethra at a less obtuse angle than those from other zones, thus potentially making them more prone to the reflux of urine, perhaps with a contribution from turbulence [154]. This anatomy makes the peripheral zone more susceptible to exposure from infectious agents [155,156] and xenobiotics, either environmental or plant-derived bioactives, and their phase I/II metabolites, that may be present in the urine and have the potential to either enhance or reduce risk of PCa. The higher incidence of PCa (70%–80% of all PCas) within the peripheral zone compared to other zones may be a consequence of this exposure [6], in addition to an increased risk of PCa progression due to genomic alterations [157,158,159]. It is therefore plausible that the prostate is exposed to plant-derived bioactive compounds present in urine, and in this way, may exert their potential beneficial effects via their anti-microbial, anti-inflammatory and immunomodulatory mechanisms, as described above.

There is also increasing evidence for a microbiological community associated with both the urinary tract and the prostate itself [160,161]. While certain members of this community, linked to both urinary tract and prostatic infections, may increase the risk of malignant changes [162,163,164,165], others (of particular relevance to the sulfur metabolites present in cruciferous and alliaceous vegetables) may metabolise plant-derived chemicals either from systemic exposure or from urinary reflux, to more biologically active forms [26,57].

## 6. Future Perspectives

Health claims pertaining to nutrition are highly regulated in order to inform as well as protect the public from false health declarations. In order to substantiate scientific findings, human intervention studies, although challenging, are therefore essential in supporting the role of plant-derived bioactives in preventing PCa [166,167]. Central to this is the careful consideration that needs to be given to experimental design, selection of appropriate biomarkers of effect and/or clinical endpoints, as well as defining the appropriate interventions [167]. For example, it may be difficult to extrapolate concentrations from those used in vitro to clinical dosages for use in human studies. Understanding of the bioavailability and nutrikinetics of relevant phytochemicals is therefore essential to the effective design and outcome of human trials [81,148,168,169]. These factors will inevitably alter dependent upon practicalities such as their development and route of administration.

One option for determining the effects of individual plant-derived bioactives is to deliver pure compounds through dietary supplements. However, isolating pure compounds is sometimes challenging due to issues with stability, but also the potential to miss, in some cases, unknown compounds derived from the same plant of interest that may also exhibit bioactivity either additively or synergistically [81]. Another option, either individually or in combination with dietary supplements, is to utilise genomic technologies and develop novel foods that will deliver increased bioactives whilst maintaining the relatively unchanged background composition of the plant [170]. Such novel foods would not only differentiate between the effects mediated by individual bioactives compared to effects due to the ingestion of the whole plant, but also facilitate double-blinded intervention trials, the gold standard in clinical nutrition. One such example is the development of the high-GRN broccoli [45]. However, the development of such novel foods requires significant investment of both time and effort to understand and manipulate plant biosynthetic pathways.

An alternative would be to utilise knowledge on levels of individual plant-derived bioactives across different plants/foods to select appropriate interventions. There are a handful of comprehensive and searchable databases with up-to-date coherent and validated scientific information on the composition of food-derived bioactives. The three core databases which provide extensive data on the composition of bioactive compounds in foods are: USDA Database for the Flavonoid Content of Foods Release 3.3 [171], Phenol-Explorer polyphenol content in foods [172], and eBASIS (Bioactive Substances in Food Information System) [173]. The former two are concerned predominantly with dietary flavonoids and polyphenols. eBASIS includes both the composition and beneficial bioeffects of polyphenols, as well as additional bioactive compound classes of relevance to PCa, including glucosinolates and curcuminoids. 

The eBASIS database is a collation of data relating to bioactive compounds in commonly consumed plant-based foods such as fruits as well as cruciferous and alliaceous vegetables. The database is divided into two principal sections: composition data and bioeffects data. The entire database includes data from over 1300 peer-reviewed publications, forming 44,000 data points, covering 800 compounds in over 270 food plants, with the data linked to authoritative plant and plant-part lists [174,175,176]. The use of comprehensive databases is essential to improve the estimation of dietary intake levels and to study the nature and dose–activity relationships of their biological effects.

## 7. Conclusions

Many plant bioactives have been shown to exhibit multi-modal effects on PCa cells (Figure 10), and foods such as cruciferous and alliaceous vegetables, tomatoes, red wine, green tea, turmeric and pomegranate have all been linked with a reduced PCa risk through epidemiological studies. The accurate assessment of dietary intake through methods such as food frequency questionnaires and food diaries remains challenging [81]. In addition, although PSA may act as a surrogate for disease activity [177], its use as a biomarker for PCa diagnosis is often difficult to interpret during the early stages of PCa development [178,179]. Due to the heterogenous nature of PCa progression [180], there is a need for better assays with increased sensitivity and specificity, which could be used to evaluate the role of plant bioactives in PCa prevention in human intervention studies. Further insights into the role of individual foods and their chemical components, as well as dietary patterns, in preventing the occurrence of aggressive PCa will need to come from well-designed human intervention studies.

## Figures and Tables

**Table 1:** Summarised epidemiological and human studies for the dietary bioactives discussed. ADT: androgen deprivation treatment; PCa: prostate cancer; PSA: prostate-specific antigen; SFN: sulforaphane; ITC: isothiocyanate; FFQ: food frequency questionnaire

Author, Year	Study Type	Patient Cohort/Intervention	Analysis
**Glucoraphanin and Sulforaphane from Cruciferous Vegetables**			
Liu et al. 2012 [39]	Meta-analysis 6 case-control 7 cohort studies	10 studies USA/Canada, 1 Asia, 2 Europe.	RR = 0.90; 95% CI 0.85–0.96 for overall cruciferous vegetable intake RR = 0.79; 95% CI 0.69–0.89 for case control studies
Richman et al. 2012 [40]	Prospective study *n* = 1560	USA PCa registry. Biopsy-verified localised PCa. Clinical survey and FFQ at baseline and every 6 months	59% reduced risk of PCa progression for highest vs. lowest intake of cruciferous vegetables. HR: 0.41, 95% CI 0.22–0.76 (*p* = 0.003)
Zhang et al. 2019 [44]	2-arm parallel randomised double-blinded intervention trial *n* = 98	USA cohort. Intervention for 4-6 weeks prior to prostate biopsy procedure	Accumulation of urine and plasma SFN ITCs and individual SFN metabolites. 40 differentially expressed genes correlated with treatment Downregulation of *AMACR* and *ARLNC1* genes No significant difference in HDAC activity or prostate tissue biomarkers
Traka et al. 2019 [45]	3-arm parallel randomised double-blinded 12-month intervention trial *n* = 49	UK cohort. Prostate biopsies at the start and end of 12-month intervention	Dose-dependent attenuation of gene expression and associated oncogenic pathways
**SACSO from Alliaceous Vegetables**			
Hsing et al. 2002 [53]	Population based study 238 case subjects 471 control subjects	Shanghai, China. Cases: histologically confirmed PCa. In-person interviews and FFQ.	Highest allium intake (>10.0 g/day) OR = 0.51, 95% CI 0.34–0.76 *p* < 0.001 Garlic (OR = 0.47, 95% CI 0.31–0.71; *p* < 0.001)
Zhou et al. 2013 [54]	Systematic literature review 6 case-control 3 prospective cohort studies	3 studies Europe, 3 studies USA, 2 studies Asia, 1 Australia. Interview or self-administered FFQ	OR = 0.82 95% CI 0.70–0.97 for allium intake OR = 0.77 95% CI 0.64–0.91 for garlic intake
**Lycopene from Tomatoes**			
Rowles et al. 2017 [77]	Systematic literature review 43 case control studies	32 studies N. America, 6 Europe, 2 Australia, 2 Asia (China and Singapore), 1 S. America. FFQs and 1 x interview	RR = 0.88, 95% CI 0.78−0.98, *p* = 0.017 (for localised PCa risk) RR = 0.88, 95% CI 0.79−0.98, *p* = 0.019 for circulating lycopene concentrations (and localised PCa risk) Dose-response seen. No effect for risk of advanced PCa.
Wang et al. 2015 [78]	Systematic review and dose-response meta-analysis 10 cohort 11 nested case-control 13 case-control studies	22 studies N. America, 7 studies Europe, 2 Australia, 2 Asia, 1 S. America	RR = 0.86, 95% CI 0.75–0.98 (localised PCa risk) RR 0.81, 95% CI 0.69–0.96 for blood lycopene levels. Dose-response seen No effect for risk of advanced PCa
Van Hoang et al. 2018 [79]	Case-control study *n* = 652	Vietnamese cohort. Cases (244) with localised PCa, and PSA 4 ng/mL. Face-to-face interviews using a structured semi-quantitative validated FFQ	OR = 0.46 95% CI 0.27–0.77 for highest lycopene intake
Key et al. 2015 [80]	Pooled Analysis of 15 studies 15 case-control studies *n* = 29780	6 studies Europe, 6 studies US, 1 study Afro-Caribbean, 1 Australia, 1 Mixed-cohort (Australia and Europe)	No association between intake and overall risk of PCa. OR 0.65 95% CI: 0.46, 0.91; *p* = 0.032 for risk of advanced stage PCa and aggressive disease
Giovannucci et al. 2002 [82]	Prospective Study *n* = 47365	US male health professional cohort – ‘Health Professionals Follow-Up Study’ (HPFS)	RR = 0.84 CI 0.73–0.96 *p* = 0.03 for total intake RR 0.77 95% CI 0.66–0.90 *p* < 0.001 for high tomato sauce intake RR = 0.65 95% CI 0.42–0.99 for high tomato sauce intake and extra-prostatic cancers
Kim et al. 2003 [83]	Double-blinded 2-arm randomised control trial *n* = 32	US cohort. Tomato sauce intervention vs. no intervention for 3 weeks prior to prostatectomy	Increased abundance of apoptotic cells (from 0.84 +/- 0.13% to 2.76 +/- 0.58% *p* = 0.0003) and degree of apoptotic cell death (from 0.84 +/- 0.13% to 1.17 +/- 0.19% *p* = 0.028) in resected tumour areas
**Resveratrol from Wine**			
Vartolomei et al. 2018 [99]	Meta-analysis 17 studies *n* = 611169 Meta-analysis of moderate wine intake: 14 studies *n* = 455413 6 cohort 8 case control studies	4 studies Canada, 4 studies Europe, 4 studies USA, 2 studies Australia	No increased risk of PCa 0.98 95% CI 0.92–1.05, *p* = 0.57 (for all wine) Increased risk of PCa with moderate intake of white wine (RR 1.26 95% CI 1.10–1.43 *p* = 0.001) Decreased risk of PCa with moderate intake of red wine RR 0.88 95% CI 0.78–0.999 *p* = 0.047 (risk reduction of 12%)
**Catechins from Green Tea**			
Guo et al. 2017 [109]	Systematic review and meta-analysis: 7 observational: 4 cohort 3 case control 3 randomised controlled trials	6 studies Asia (incl. 1 Singapore, 4 Japan, 1 China), 2 Europe, 1 N. America, 1 Africa	RR 0.75 95% CI 0.53–1.07 for highest versus lowest category of green tea intake Dose response with each 1 cup/day increase of green tea 0.954 (95% CI 0.903–1.009) *p* = 0.08 after removal of heterogeneity and undertaking sensitivity analysis RR of 0.38 (95% CI 0.16–0.86, *p* = 0.02) - Patients with high-grade prostatic intraepithelial neoplasia (HGPIN) or atypical small acinar proliferation (ASAP)
**Curcumin from Turmeric**			
Choi et al. 2019 [126]	Randomised double-blind, placebo-controlled trial *n* = 82	S. Korean cohort. Curcumin capsule intervention or placebo from the beginning of ADT withdrawal.	PSA progression: 10.3% (treatment) vs 30.2% (control) *p* = 0.0259
Hejazi et al. 2016 [127]	Randomised double-blind, placebo-controlled trial *n* = 40	Iranian cohort. Curcumin capsule intervention or placebo during external-beam radiotherapy.	Increase in plasma total antioxidant capacity (TAC) significantly higher in the treatment arm (*p* < 0.001) Reduction in activity of superoxide of superoxide dismutase (SOD) in the treatment arm (*p* = 0.026)
**Ellagitannins from Pomegranate**			
Pantuck et al. 2006 [144]	Phase II two-stage clinical trial *n* = 46	US cohort. PSA 0.2–5ng/mL documented as rising. 8 ounces of pomegranate juice daily (570 mg total polyphenol gallic acid) until disease progression	Increase in mean PSA doubling time significantly increased with treatment: 15 months at baseline to 54 months post-treatment (*p* < 0.001)
Paller et al. 2013 [145]	Randomised phase II study *n* = 104 (*n* = 92 for analysis)	US cohort (multi-centre). Rising PSA without evidence of metastasis. 1g vs. 3g pomegranate extract capsules daily for up to 18 months.	Increase in PSA doubling time with treatment: 11.9 months at baseline to 18.5 months post-treatment (*p* < 0.001)
Stenner-Liewen et al. 2013 [146]	Randomised placebo-controlled trial *n* = 109 (advanced PCa)	Swiss cohort. PCa with PSA 5ng/mL. 500mL pomegranate juice vs. placebo beverage daily for 4 weeks, then all 250mL POM juice for 4 weeks. PSA measured at defined timepoints.	No significant difference in PSA levels at 28 days (*p* = 0.11)
Freedland et al. 2013 [147]	Randomised double-blind placebo-controlled trial. *n* = 69	US cohort. Pomegranate extract capsules or placebo for up to 4 weeks prior to prostatectomy	Urolithin A accumulation (*p* = 0.031) No effect on oxidative stress / proliferation / progression / PSA levels

## Abbreviations

ACC1	acetyl-CoA carboxylase 1
ADT	androgen deprivation treatment
ASAP	atypical small acinar proliferation
AR	androgen receptor
BCG	Bacillus Calmette-Guerin
Bcl-2	B-cell lymphoma 2
Bcl-xL	B-cell lymphoma extra-large
COX-2	cyclooxygenase-2
CBP	cAMP response element-binding protein
CPT1A	carnitine palmitoyltransferase 1A
CVD	cardiovascular disease
CXCR4	chemokine receptor type 4
DAS	diallyl sulfide
DADS	diallyl disulfide
DATS	diallyl trisulfide
DHEA	dehydroepiandrosterone
eBASIS	Bioactive Substances in Food and Information System
EC	epicatechin
ECG	epicatechin-3-gallate
EGC	epigallocatechin
EGCG	epigallocatechin-3-gallate ERK1/2: extracellular signal-regulated protein kinase 1 and 2
FA	fatty acid
FASN	fatty acid synthase
FOXO	forkhead family of transcription factors
GRN	glucoraphanin
GM	gut microbiota
GSL	glucosinolate
GST	glutathione-S-transferase
GSTM1	Glutathione S-Transferase Mu 1
HDAC	histone deacetylases
HGPIN	high-grade prostatic intraepithelial neoplasia
HLH	helix-loop-helix
IAD	intermittent androgen deprivation
IARC	International Agency for Research on Cancer
Id1	inhibitor of DNA binding 1
IGF	insulin-like growth factor
Keap1	kelch-like ECH-associated protein
ITC	isothiocyanate; MDM2: mouse double minute 2 homolog
MEK	mitogen-activated protein kinase kinase
MMPs	matrix metalloproteinases
MMTSI	S-methyl methanethiosulfinate
MMTSO	S-methyl methanethiolsulfinate
MTA1	metastasis-associated protein 1
NADPH	nicotinamide adenine dinucleotide phosphate
NF-κB	nuclear factor-kappa B
Nrf-2	nuclear factor erythroid 2-related factor 2
OSC	organosulfur compounds
PCa	prostate cancer
PI3K	phosphoinositide 3-kinase
PKB/Akt	protein kinase B
PSA	prostate specific antigen
RCT	randomised controlled trial
ROS	reactive oxygen species
SACSO	S-alk(en)yl-L-cysteine sulfoxide
SMCSO	S-methyl-L- cysteine sulfoxide
SCMCSO	S-carboxymethyl-L-cysteine sulfoxide; SFN: sulforaphane
SFN-Cys	sulforaphane-cysteine
SFN-NAC	sulforaphane-N-acetyl-cysteine
TRAIL	TNF-related apoptosis inducing ligand
TRAMP	transgenic adenocarcinoma of the mouse prostate
TPB	template-guided transperineal prostate biopsy
VEGF	vascular endothelial growth factor
VEGFR2	vascular endothelial growth factor receptor 2
